# Assessment of Hand Function Through the Coordination of Contact Forces in Manipulation Tasks

**DOI:** 10.2478/hukin-2013-0001

**Published:** 2013-03-28

**Authors:** Slobodan Jaric, Mehmet Uygur

**Affiliations:** 1 Department of Kinesiology and Applied Physiology, University of Delaware, Newark, USA.; 2 Biomechanics and Movement Science Graduate Program, University of Delaware, Newark, USA.

**Keywords:** grip force, load force, coupling, scaling, modulation

## Abstract

Exploration of force coordination has been one of the most often used approaches in studies of hand function. When holding and manipulating a hand-held object healthy individuals are typically able to highly coordinate the perpendicular (grip force; GF) with the tangential component of the contact force (load force; LF). The purpose of this review is to present the findings of our recent studies of GF-LF coordination. Regarding the mechanical factors affecting GF-LF coordination, our data suggest that both different hand segments and their particular skin areas could have markedly different friction properties. It also appears that the absolute, rather than relative safety margin (i.e., how much the actual GF exceeds the minimum value that prevents slipping) should be a variable of choice when assessing the applied magnitude of GF. The safety margin could also be lower in static than in free holding tasks. Regarding the involved neural factors, the data suggest that the increased frequency, rather than an increased range of a cyclic LF could have a prominent detrimental effect on the GF-LF coordination. Finally, it appears that the given instructions (e.g., ‘to hold’ vs. ‘to pull’) can prominently alter GF-LF coordination in otherwise identical manipulation tasks. Conversely, the effects of handedness could be relatively week showing only slight lagging of GF in the non-dominant, but not in the dominant hand. The presented findings reveal important aspects of hand function as seen through GF-LF coordination. Specifically, the use of specific hand areas for grasping, calculation of particular safety margins, the role of LF frequency (but not of LF range) and the effects of given instructions should be all taken into account when conducting future studies of manipulation tasks, standardizing their procedures and designing routine clinical tests of hand function.

## Introduction

Human hand is a ‘tool’ routinely used to interact with almost all objects in our physical environment. It can perform a wide variety of actions from brittle and gentle, such as feeding, carving a tool and caressing, to a heavy labor, such as using a tool to fight or to lift heavy objects. A skilled use of hands can be considered as the most important among our motor abilities since marked impairment of the hand function brings the heaviest burden to the daily life. Specifically, even a mild dysfunction in the control of manipulative actions seriously affects one’s independent living. Therefore, the hand function has been studied through a variety of approaches, such as behavioral, kinematic, kinetic, electromyographic etc. Each of those approaches gives its specific insight into the neural control of manipulative actions. Within this study, we selected the kinetic approach which can be based on a frequently used simple model of mechanical interaction between the hand and the hand-held object. Moreover, the kinetic variables routinely used in the studies of hand function have proven to be valid measures of mechanical and control properties of manipulative actions (Johansson and Westling, 1984; de Freitas et al., 2008a). Finally, note that the same variables proved to be sensitive to detect the differences in hand function between healthy subjects and the individuals known for impaired hand function, such as the neurological patients (Nowak and Hermsdorfer, 2006; Krishnan et al., 2008; Krishnan and Jaric, 2008).

### Mechanics and neural control of manipulation

An object can be manipulated in a variety of ways depending on task requirements. For example, one can apply a precision grip (i.e., only the finger tips are in the contact with the object) while holding a champagne glass, a power grip (entire ventral part of the hand is used) when using a hammer, or a two-hand power grip when manipulating large and heavy objects. Regardless of the grip type, one has to apply a certain magnitude of force perpendicularly to the grasping sides of the object to prevent a slippage caused by tangential forces that can originate either from the object’s weight and inertia, or from external reaction forces. The perpendicular (i.e., normal) force applied against the object surface will be referred to as grip force (GF), while the tangential force acting in parallel to the object’s contact surface will be referred to as load force (LF).

According to a routinely used simple mechanical model of holding a vertically oriented object (Johansson and Westling, 1984), the minimum GF (GF_min_) has to be at least equal to the ratio between LF and the static coefficient of friction (COF) between the digits and the object surface in order to prevent slippage:

(eq. 1) $${\mathrm{GF}}_{\min}=\mathrm{LF}/\mathrm{COF}.$$

However, during object manipulations individuals inevitably apply GF that is somewhat higher than GF_min_. Either the absolute or relative difference between the employed GF and GF_min_ required to prevent slippage (GF-GF_min_) has been referred to as the safety margin. It has been shown that the safety margin remains relatively low and stable during object manipulation even when LF is rapidly changing (Johansson and Westling, 1984; Westling and Johansson, 1984).

Keeping a low safety margin has been interpreted as a buffering strategy that keeps GF high enough to maintain stability and prevent accidental slipping of an object and yet keeps GF low enough to prevent both the associated muscle fatigue and crushing the object due to excessive forces. As a result, it has been concluded that the CNS closely monitors the changes in the LF and coordinates the GF in an anticipatory fashion with the LF while performing various manipulation tasks (Johansson and Westling, 1988; Flanagan and Wing, 1995).

### Variables used to assess GF-LF coordination

A variety of dependent variables have been used to evaluate the GF-LF coordination in various manipulation tasks. The indices of GF-LF coordination routinely assessed in different tasks have been GF scaling, GF-LF coupling, and GF modulation. Regarding the GF scaling, the GF to LF ratio (GF/LF ratio) has been calculated as the ratio of either the average or peak values of GF and LF. Elaborate GF-LF coordination has been mostly associated with a relatively low and stable GF/LF ratio even during a rapidly changing LF (Johansson and Westling, 1984; de Freitas et al., 2007). Another frequently employed index of GF-LF coordination has been usually referred to as GF-LF coupling. It has been evaluated by the maximum cross-correlation coefficient and the corresponding time lag observed between GF and LF time series (Flanagan and Wing, 1995; Jaric et al., 2006; Danion et al., 2009; de Freitas and Jaric, 2009; Danion et al., 2010). A high coupling has been seen as both the high values of the correlation coefficients observed between GF and LF time series and the time lag between them close to zero. Lastly, GF modulation shows how much GF adapts to the ongoing changes in LF. GF modulation has been routinely assessed through the regression lines obtained from the GF-LF diagrams, where the slope of the regression line represents the GF gain, while the intercept represents the GF offset (Flanagan and Wing, 1995; de Freitas and Jaric, 2009). Both a high value of GF gain and a low value of GF offset have been considered as indices of high GF-LF coordination.

All of the above mentioned indices of GF-LF coordination might show dependency on numerous factors, such as various task variables and conditions, the populations tested and so on. For example, neurological patients (e.g., individuals with multiple sclerosis, cerebral palsy, Parkinson’s disease, and stroke) consistently reveal disrupted force control during object manipulation that usually results in an elevated GF/LF ratio (Nowak and Hermsdorfer, 2005; Krishnan et al., 2008; Krishnan and Jaric, 2008; Mackenzie et al., 2009). Therefore, the studies of GF-LF coordination have been seen as a promising approach for development of quantitative clinical tests of hand function (Jaric et al., 2005a; Nowak and Hermsdorfer, 2006; Krishnan and Jaric, 2008). Aside from the neurological populations, a deteriorated GF-LF coordination has been seen in healthy individuals during performance of presumably complex and demanding tasks, such as when the applied LF continuously changes its direction (Jaric et al., 2005b; de Freitas et al., 2007; Freitas et al., 2007; de Freitas et al., 2008b), when the frequency of LF change is particularly high (e.g., when shaking an object or producing an oscillatory LF against an external support (Flanagan and Wing, 1995; Jaric et al., 2006)), when the actions of two hands are dissimilar (Serrien and Wiesendanger, 2001; Krishnan and Jaric, 2010), or when the visual feedback (Danion et al., 2010) or gravitational field (White et al., 2005) is altered. Although most of these phenomena have been extensively studied, the role of a number of other potentially important mechanical and neural factors still remains largely unexplored. Within the following sections we will present our recent studies aimed towards exploring the role of a several presumably mechanical and neural factors that could affect GF-LF coordination. The expected findings could not only serve for further standardization of the experimental protocols aimed to explore GF-LF coordination, but also for revealing important aspects of neural control of manipulative actions.

### Mechanical Factors: Friction

Depending on particular requirements of a manipulation task, we use different grasping techniques that involve not only different hand segments, but also their various skin areas. For example, the pinch and precision grasp (commonly used during precise manipulation of light objects, such as picking up a coin or grasping a champagne glass) involve the tips of the digits and the thumb, while the uni- and bi-manual power grasps (commonly used during manipulating heavy objects, such as holding a tool or carrying a box) involve large ventral areas of the digits and the palm. When the hand specialization for manipulative actions is considered, one could speculate that the frictional properties of the hand areas that are commonly used during object manipulations (i.e. “specialized” hand areas) might have higher COF than the other hand areas that are not commonly used (i.e. “non-specialized” hand areas). Note that according to the above presented simple model of manipulation (see eq. 1), higher COF allows for a lower GF, which in turn causes less fatigue and allows a better control of the manipulated object (Claudon, 2006; Laroche et al., 2007).

**Figure 2:**
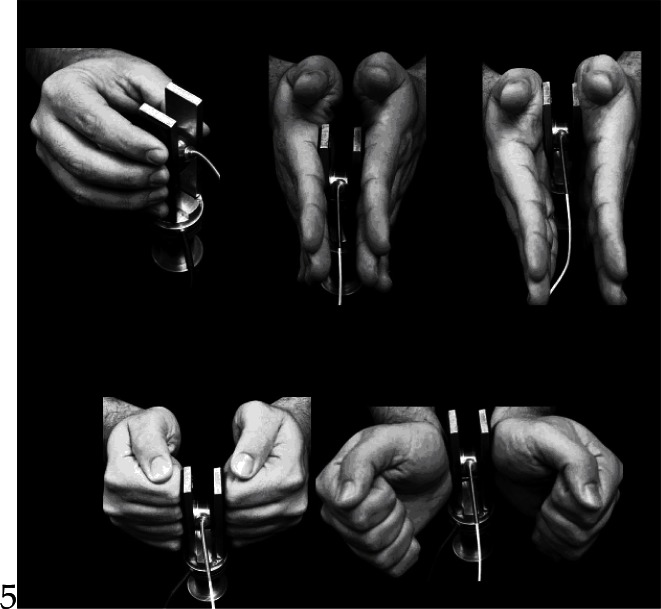
Illustration of the grasping techniques that involve ‘specialized’ (precision, distal palm and proximal palm grasps) and ‘non-specialized’ (wrist and fist grasp) segments and skin areas in the contact with the object

In our recent study we explored various grasping techniques that involve a large number of either specialized (e.g. finger tips, distal and proximal palm) or non-specialized (e.g. fist, wrist) hand areas (Figure 2) and measured the static COF of the hand areas involved in those techniques ((Uygur et al., 2010b); here we present the data from only few out of a larger sample of grasping techniques). Moreover, to test whether COF of different hand areas are also coating specific, we used both rubber and acetate coatings to represent surfaces with high and low COF, respectively. Finally, we used a standard ‘slip point’ method to calculate COF from GF and LF recorded at the instant of slip (Westling and Johansson, 1984; Savescu et al., 2008). Specifically, subjects were instructed to hold the vertically oriented object and to slowly reduce the GF until the object slips. At the time point just prior to the initiation of slipping, we calculated the ratio between the measured GF and the weight of the handle (i.e. slip ratio) and used it to calculate the static COF as:

(eq. 2) COF=1/(2 slip ratio) .

**Figure 3:**
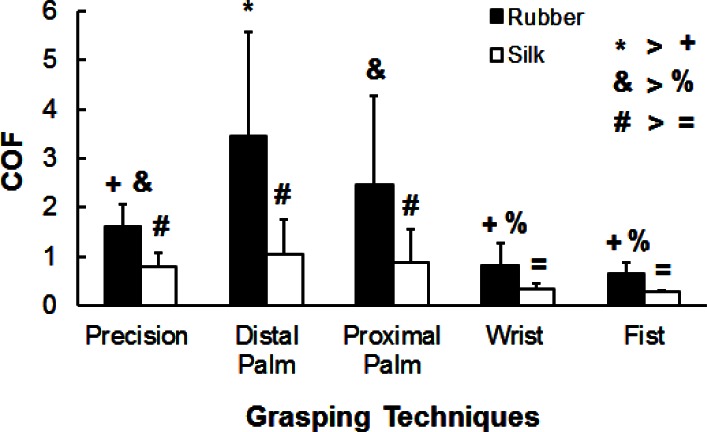
Coefficients of friction (COF; means with SD bars) obtained from different grasping techniques separately for a high friction (i.e., rubber) and low friction (silk) coating materials

Overall, our results revealed that the COF measured from the grasping techniques that involved “specialized” hand areas were higher than those measured from the grasping techniques that involved “non-specialized” hand areas (Figure 3). A higher COF is advantageous especially during heavy object manipulation since it requires less GF and, therefore, reduces the fatigue in the GF producing muscles (Seo et al., 2008) and decreases risk of hand injuries (Laroche et al., 2007). Moreover, the results revealed that the difference among the hand segments could be coating specific. Another important finding of our study is a high across subject variability of COF obtained from the same skin areas. Overall, the differences found in COF across the hand segments and coatings, as well as a high inter-subject variability of the measured COF strongly emphasizes the importance of routine assessment of COF in the future biomechanical, motor control and ergonomic studies of manipulation activities.

### Mechanical Factors: Grasping Techniques and Safety Margin

As already described, when we manipulate objects we routinely employ higher GF than the minimum required. This “excess” grip force has often been referred to as safety margin. Previous research has shown that the safety margin is one of the factors that could be closely monitored by the CNS to provide a relatively low and stable LF (Johansson and Westling, 1984; Westling and Johansson, 1984). The studies of manipulation activities have calculated safety margin either as a relative (i.e. SM_rel_; the difference between the applied GF and the GF_min_ in percentage of the GF_min_) or absolute (i.e. SM_abs_; the absolute difference between the applied GF and GF_min_ calculated in N) without exploring their particular properties (Flanagan and Wing, 1995; Jenmalm et al., 1998; Cole et al., 1999; Mrotek et al., 2004). As a consequence, it still remains unknown whether the CNS keeps either SM_rel_ or SM_abs_ invariant across a variety of static and dynamic manipulation tasks, such as those performed with the objects of different frictional properties and manipulated by using different grasping techniques.

**Figure 4:**
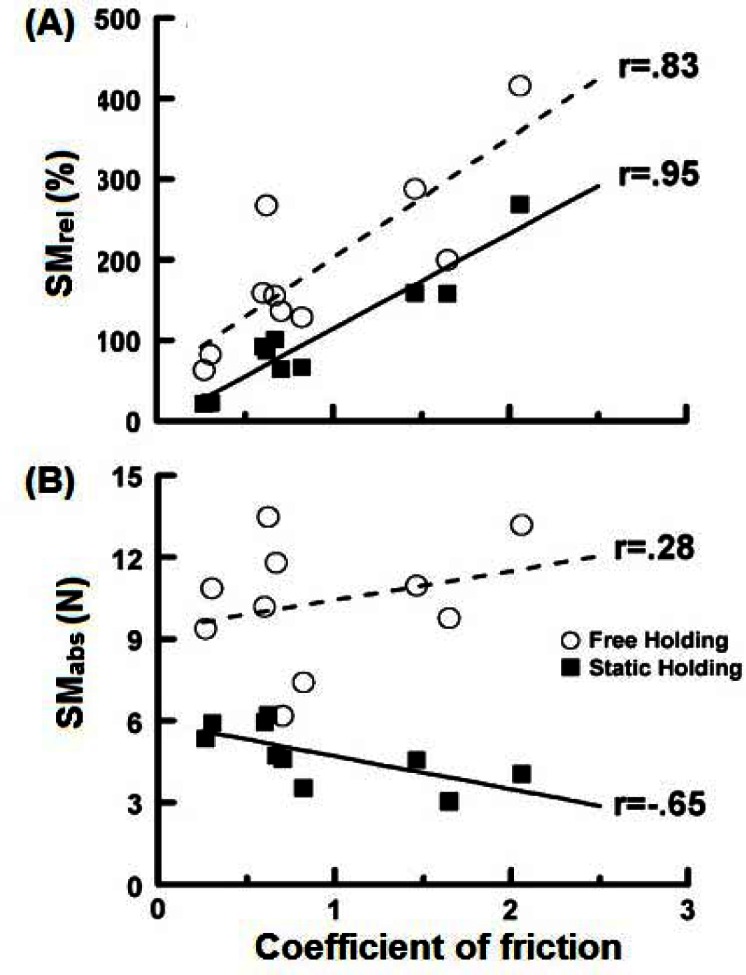
Relative (SM_rel_; A) and absolute (SM_abs_; B) safety margin as a function of the acting coefficient of friction. The dashed and solid lines correspond to the free and static holding task, respectively

We asked healthy young individuals to perform both static and free holding tasks that required exerting the same pulling force (de Freitas et al., 2009). They completed each task under five different grasping conditions (Figure 2) and two different object coatings that provided low and high coefficient of friction (e.g. acetate and rubber, respectively). We specifically analyzed both SM_rel_ and SM_abs_ as a function of friction since their values depend on the GF_min_ which is inversely proportional to the acting COF (see eq. 1). The results revealed a high and positive relationship of SM_rel_ with the acting COF in both free and static holding tasks (Figure 4A). On the other hand, SM_abs_ revealed no significant relationship with the acting COF in static holding tasks, while a moderately negative relationship was found between SM_abs_ and friction in free holding tasks (Figure 4B). Finally, a lower safety margin was observed in the static than in the free holding condition

Collectively, our results suggest that the CNS could keep SM_abs_, rather than SM_rel_ as partly invariant among the studied free and static manipulation tasks. Since SM_abs_ was found to be an invariant characteristic of GF control, we suggest that the future studies of hand function should use SM_abs_ not only when assessing the basic properties of hand function, but also when comparing hand function between healthy individuals and clinical populations. The lower safety margin observed in the static holding task could be interpreted as an acceptance of a higher risk of slipping. Namely, a partial slipping of the hand along an externally fixed object could not have such undesirable consequences as a dropping of the object in a free holding task.

### Neural Factors: LF range and frequency

Humans frequently perform continuous manipulation movements, such as shaking an object vertically or horizontally, using a hammer repetitively to strike a nail, or using an external support to maintain balance during a bus ride. These tasks are presumably controlled by feed-forward neural mechanisms requiring minimal corrections triggered by the sensory information (Flanagan and Wing, 1993; Sternad et al., 2000). To study continuous object manipulations, researchers have commonly used either dynamic tasks (e.g., cyclic arm movements with a hand-held load (Flanagan and Wing, 1995)), or static tasks (e.g., exerting an oscillatory force against an externally fixed device (Jaric et al., 2005a; Jaric et al., 2005b; Jaric et al., 2006; de Freitas and Jaric, 2009; Uygur et al., 2012)). Overall, the results have revealed that an increase in the frequency of an oscillatory task could be associated with a deteriorated GF-LF coordination (Flanagan and Wing, 1995; Jaric et al., 2006). However, this deterioration might not have been caused by the frequency itself, but rather by the increase in the rate of LF change. An oscillatory pattern of LF can be modeled as a sinusoidal function:

(eq. 3) $$LF={LF}_{0}\text{sin}\left(\omega t\right),$$

where LF_0_ is LF amplitude, while ω is the angular frequency of LF change.

Therefore, the rate of LF change is

(eq. 4) $$dLF/dt={LF}_{0}\omega \text{cos}\left(\omega t\right).$$

The last equation shows that the rate of LF change can increase not only due to an increase in the LF frequency, but also due to an increase in LF range. Keeping in mind the kinetic generalization of the minimum jerk hypothesis (i.e., when controlling voluntary movements the CNS attempts to minimize the force change (Uno et al., 1989)), one could argue that the CNS tends to minimize the GF change in the tasks that involve a high rate of LF change. A reduced GF change would inevitably lead to a decreased GF modulation and GF-LF coupling, as well as to an increased GF/LF ratio. These changes have been observed in tasks that require a high frequency of LF change (Flanagan and Wing, 1995; Jaric et al., 2006). Therefore, based on the minimum jerk hypothesis and its kinetic generalization, one could hypothesize that an increase in either LF frequency or LF range would result in a decreased GF-LF coordination.

**Figure 5:**
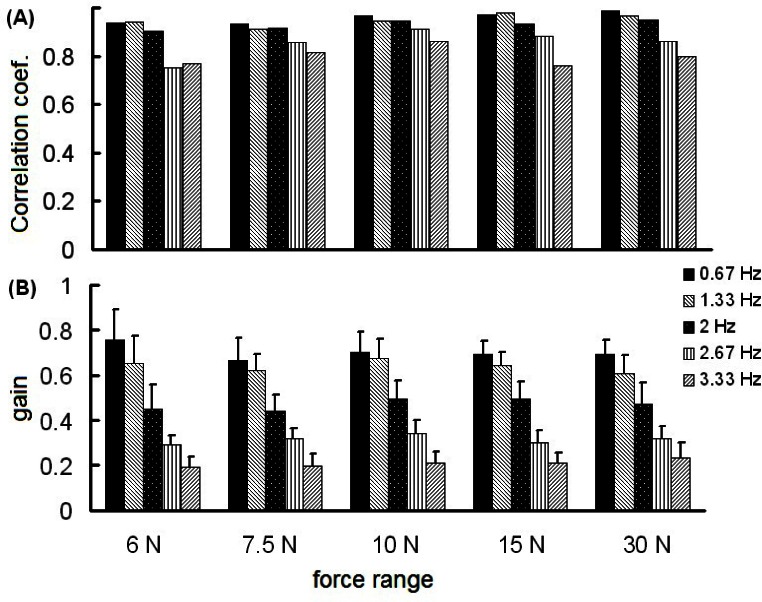
GF-LF coordination assessed from static manipulation tasks performed across different frequencies (0.67–3.33 Hz) and force ranges (6–30 N). The panels illustrate the averaged across the subjects data regarding the GF-LF coupling (i.e., the median correlation observed between GF and LF; A) and GF gain (slope of the GF-LF regression lines; B)

We asked healthy individuals to exert isometric LF profiles in an oscillatory fashion at 5 different frequencies (i.e., 0.67, 1.33, 2, 2.67, and 3.33 Hz) and within 5 different LF ranges (6, 7.5, 10, 15 and 30 N (Uygur et al., 2010a)). The results revealed a prominent effect of an increase in the LF frequency, but not an increase in LF range, on the coordination between GF and LF. Specifically, GF-LF coupling assessed through the correlations between the GF and LF time series decreased with LF frequency, but not with LF range (Figure 5A). The following panel depicts a similar finding regarding GF modulation (Figure 5B). Namely, GF gain decreases with LF frequency, but not with GF range. The lack of the effect of LF range apparently contradicts the predictions drawn from the kinetic generalization of the minimum jerk hypothesis. One could interpret these findings by the different synergies of GF and LF that could be applied in the phases of the increasing and decreasing LF and GF forces. Therefore, the future studies of hand function should consider task frequency, rather than the force range, when either designing rhythmic manipulation tasks performed under static conditions, or comparing the results of the previous research, or developing the standard tests of hand function. Future studies should explore to which extent the present findings can be generalized to the free movement tasks.

### Neural Factors: Instructions and Hand Dominance

Instructions given in various motor control experiments have been shown to affect the studied motor outcome. For example, during a pointing task that requires a discrete and accurate arm movement, the instructions to move “fast”, “fast and accurate”, and “accurate” have distinctive effects not only on the muscle neural activation, but also on the subject’s motor performance (Brown and Cooke, 1981). When the kinetic tasks are considered, giving the instructions to produce force as “*hard-and-fast as possible”* and as “*fast as possible”* revealed differences in the rate of force development, while the maximum force remained unaffected (Sahaly et al., 2001). Finally, emphasizing the action of particular muscle groups in an kinetically identical static task leads to different patterns of muscle activation (Latash and Jaric, 1998).

Regarding the role of hand dominance, it has been shown that motor lateralization could be an important factor affecting the neural control of human movements. According to the motor lateralization theory (Sainburg, 2002), the non-dominant hand could be specialized for execution of the feedback dominated tasks, while the dominant hand could be more specialized for performing the dynamic, feed-forward controlled tasks. Although previous research has partly studied the effect of handedness on GF-LF coordination of manipulation tasks (Ferrand and Jaric, 2006; de Freitas et al., 2007), the above hypothesized effect of motor lateralization has never been tested in the context of GF-LF coordination in manipulation tasks when similar movements were performed under different instructions.

**Figure 6:**
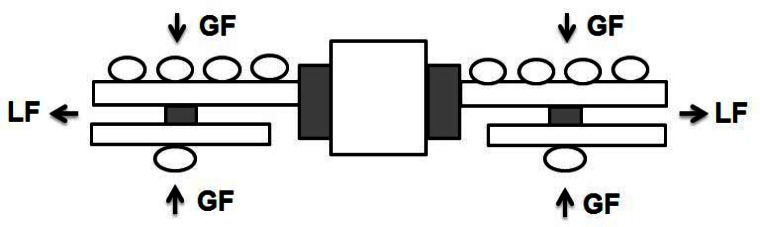
A schematic illustration of the manipulation condition. A free-moving device consists of 2 mutually attached handles and each of them records the grip (GF) and load force (LF). Circles illustrate positions of fingers and the thumb of the subject’s hands. Note that steady holding of the device requires two opposing LF to be equal

We recently conducted an experiment designed to explore the effects of both the instruction and handedness on the performance and GF-LF coordination in static bimanual manipulation tasks (Jin et al., 2011). Subjects were asked to bimanually exert an oscillatory (i.e., approximately sinusoidal) LF profile while holding stationary the system of two mechanically attached handles that was free to move (Figure 6). Since the instructions “to pull” and “to hold” have not only been routinely used in studies of manipulation actions, but also frequently swapped over without paying particular attention to them, we used three different sets of instructions. In particular, we asked the subjects: (1) to “pull” the handles equally with both hands, (2) to “pull” with right hand and to “hold” with left hand, and (3) to “hold” with right hand and to “pull” with left hand. One should keep in mind that due to the nature of the manipulation task (i.e., keeping a free moving device stationary while the hands were either pulling or holding), the exerted LF by two hands had to be the same. Therefore, the tasks were mechanically identical regardless of the given instructions.

**Figure 7:**
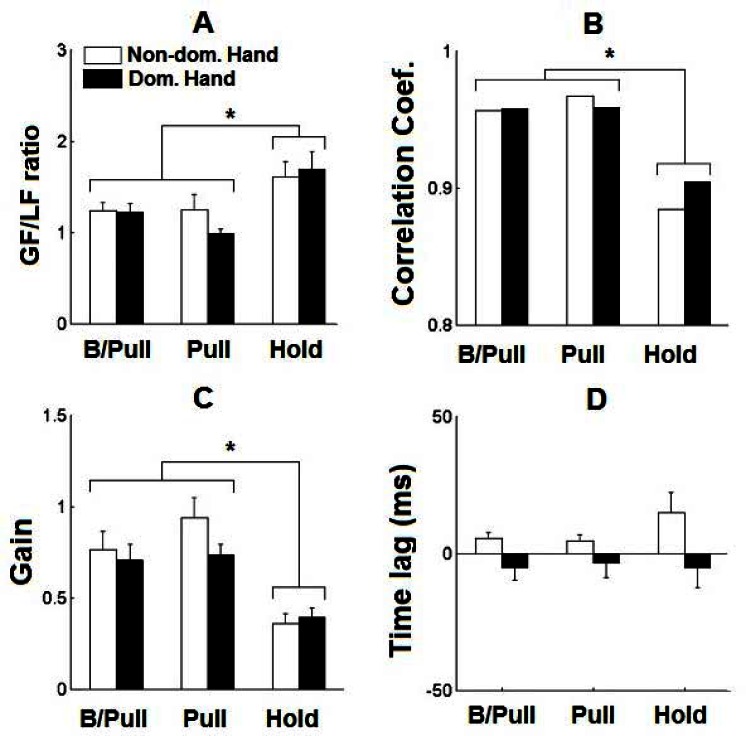
GF-LF coordination indices observed in oscillation tasks (data average data across subjects with standard error bars) depending on the given instructions to pull or to hold with particular hand, and to pull with both hands (B/Pull). Data are presented separately for the dominant and non-dominant hand. A) GF scaling assessed by the GF/LF ratio. B) GF-LF coupling assessed by correlation coefficients between them. C) GF modulation assessed by the slopes of the GF-LF regression lines. D) Time lags between GF and LF (positive values indicate lagging of GF with respect to LF)

The results revealed that the instructions used in manipulation tasks could markedly affect the coordination between GF and LF. Specifically, the instruction to “pull” revealed higher indices of GF-LF coordination through a lower GF scaling (Figure 7A), higher GF-LF coupling (Figure 7B), and higher GF modulation (Figure 7C), than the instruction to “hold”. Note that the results were similar regardless of whether both or only one hand was pulling. Regarding the handedness, the only effect we observed was a somewhat increased time lags between GF and LF in the non-dominant hand (i.e., LF leading GF) when compared to the same time lags obtained from the dominant hand (Figure 7D).

Overall, the observed findings suggest that instructions could have a prominent effect on GFLF coordination. Therefore, the instructions need to be meticulously planned in the studies of manipulation activities, particularly when asking participants “to pull” and “to hold”. Regarding the effects of handedness, note that the observed time lags were both partly inconsistent and relatively small, particularly when compared with the time needed for voluntary correction of motor acts. Nevertheless, the results suggest a partial involvement of the feedback neural mechanisms in the control of the non-dominant hand. A lack of other effects of handedness could be explained by the predominantly static nature of the tested tasks (Sainburg, 2002) and, therefore, future studies could apply a similar approach to dynamic manipulation tasks.

## Summary

Within the present paper, we reviewed the results of several of our recent studies aimed to explore the important mechanical and neural factors affecting the GF-LF coordination. All reported findings could be of profound importance for designing, as well as for interpreting and comparing the results of different studies of manipulation tasks. Taking into account that the tested tasks could be of importance for development of future standard quantitative tests of hand function, the presented material could also be of importance for standardizing their procedures.

## Figures and Tables

**Figure 1:**
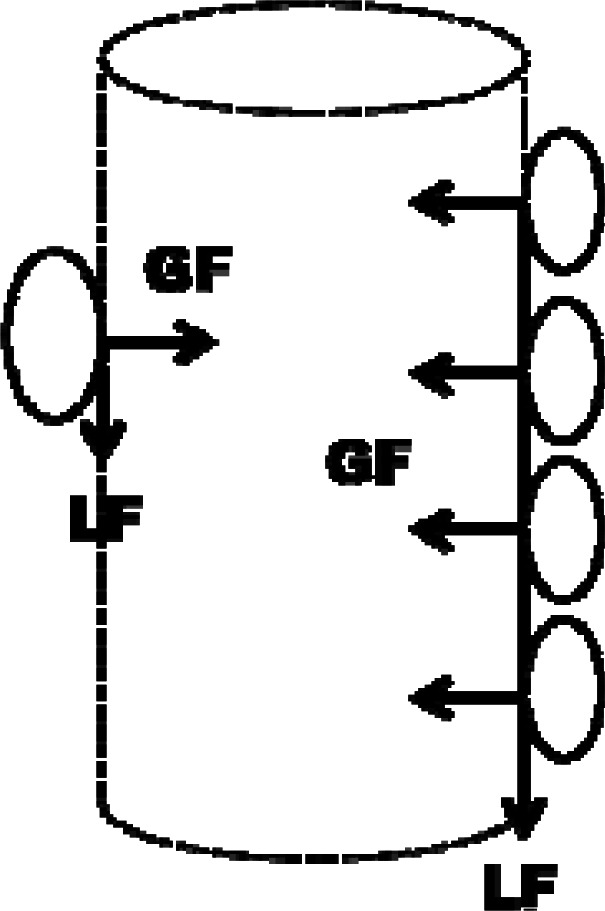
Illustration of a simple mechanical model of holding a vertically oriented object. Slippage caused by tangential force that originates from the object’s weight and inertia (load force; LF) is prevented by reaction force that originates from the perpendicular force (grip force; GF) due to the acting friction. Circles illustrate the tips of the fingers and the thumb.

## References

[b1-jhk-36-5] Brown SH, Cooke JD (1981). Amplitude- and instruction-dependent modulation of movement-related electromyogram activity in humans. J Physiol.

[b2-jhk-36-5] Claudon L (2006). Influence on grip of knife handle surface characteristics and wearing protective gloves. Appl Ergon.

[b3-jhk-36-5] Cole KJ, Rotella DL, Harper JG (1999). Mechanisms for age-related changes of fingertip forces during precision gripping and lifting in adults. J Neurosci.

[b4-jhk-36-5] Danion F, Descoins M, Bootsma RJ (2009). When the fingers need to act faster than the arm: coordination between grip force and load force during oscillation of a hand-held object. Exp Brain Res.

[b5-jhk-36-5] Danion F, Sarlegna FR, Baud-Bovy G (2010). Delayed Visual Feedback Affects Both Manual Tracking and Grip Force Control When Transporting a Handheld Object. J Neurophysiol.

[b6-jhk-36-5] de Freitas PB, Jaric S (2009). Force coordination in static manipulation tasks performed using standard and nonstandard grasping techniques. Exp Brain Res.

[b7-jhk-36-5] de Freitas PB, Krishnan V, Jaric S (2007). Force coordination in static manipulation tasks: effects of the change in direction and handedness. Exp Brain Res.

[b8-jhk-36-5] de Freitas PB, Krishnan V, Jaric S (2008a). Force Coordination in Object Manipulation. J Hum Kinetics.

[b9-jhk-36-5] de Freitas PB, Markovic G, Krishnan V, Jaric S (2008b). Force coordination in static manipulation: Discerning the contribution of muscle synergies and cutaneous afferents. Neurosci Lett.

[b10-jhk-36-5] de Freitas PB, Uygur M, Jaric S (2009). Grip force adaptation in manipulation activities performed under different coating and grasping conditions. Neurosci Lett.

[b11-jhk-36-5] Ferrand L, Jaric S (2006). Force coordination in static bimanual manipulation: Effect of handedness. Motor Control.

[b12-jhk-36-5] Flanagan JR, Wing AM (1993). Modulation of grip force with load force during point-to-point arm movements. Exp Brain Res.

[b13-jhk-36-5] Flanagan JR, Wing AM (1995). The stability of precision grip forces during cyclic arm movements with a hand-held load. Exp Brain Res.

[b14-jhk-36-5] Freitas PB, Krishnan V, Jaric S (2007). Elaborate force coordination of precision grip could be generalized to bimanual grasping techniques. Neurosci Lett.

[b15-jhk-36-5] Jaric S, Collins JJ, Marwaha R, Russell E (2006). Interlimb and within limb force coordination in static bimanual manipulation task. Exp Brain Res.

[b16-jhk-36-5] Jaric S, Knight CA, Collins JJ, Marwaha R (2005a). Evaluation of a method for bimanual testing coordination of hand grip and load forces under isometric conditions. J Electromyogr Kines.

[b17-jhk-36-5] Jaric S, Russell EM, Collins JJ, Marwaha R (2005b). Coordination of hand grip and load forces in uni- and bidirectional static force production tasks. Neurosci Lett.

[b18-jhk-36-5] Jenmalm P, Goodwin AW, Johansson RS (1998). Control of grasp stability when humans lift objects with different surface curvatures. J Neurophysiol.

[b19-jhk-36-5] Jin X, Uygur M, Getchell N, Hall SJ, Jaric S (2011). The effects of instruction and hand dominance on grip-to-load force coordination in manipulation tasks. Neurosci Lett.

[b20-jhk-36-5] Johansson RS, Westling G (1984). Roles of glabrous skin receptors and sensorimotor memory in automatic control of precision grip when lifting rougher or more slippery objects. Exp Brain Res.

[b21-jhk-36-5] Johansson RS, Westling G (1988). Programmed and triggered actions to rapid load changes during precision grip. Exp Brain Res.

[b22-jhk-36-5] Krishnan V, de Freitas PB, Jaric S (2008). Impaired Object Manipulation in Mildly Involved Individuals with Multiple Sclerosis. Motor Control.

[b23-jhk-36-5] Krishnan V, Jaric S (2008). Hand function in multiple sclerosis: Force coordination in manipulation tasks. Clin Neurophysiol.

[b24-jhk-36-5] Krishnan V, Jaric S (2010). Effects of Task Complexity on Coordination of Inter-Limb and Within-Limb Forces in Static Bimanual Manipulation. Motor Control.

[b25-jhk-36-5] Laroche C, Barr A, Dong H, Rempel D (2007). Effect of dental tool surface texture and material on static friction with a wet gloved fingertip. J Biomech.

[b26-jhk-36-5] Latash ML, Jaric S (1998). Instruction-dependent muscle activation patterns within a two-joint synergy: separating mechanics from neurophysiology. J Motor Behav.

[b27-jhk-36-5] Mackenzie SJ, Getchell N, Modlesky CM, Miller F, Jaric S (2009). Using Grasping Tasks to Evaluate Hand Force Coordination in Children With Hemiplegic Cerebral Palsy. Arch Phys Med Rehab.

[b28-jhk-36-5] Mrotek LA, Hart BA, Schot PK, Fennigkoh L (2004). Grip responses to object load perturbations are stimulus and phase sensitive. Exp. Brain Res.

[b29-jhk-36-5] Nowak DA, Hermsdorfer J (2005). Grip force behavior during object manipulation in neurological disorders: Toward an objective evaluation of manual performance deficits. Movement Disorders.

[b30-jhk-36-5] Nowak DA, Hermsdorfer J (2006). Objective evaluation of manual performance deficits in neurological movement disorders. Brain Research Reviews.

[b31-jhk-36-5] Sahaly R, Vandewalle H, Driss T, Monod H (2001). Maximal voluntary force and rate of force development in humans - importance of instruction. Eur J Appl Physiol.

[b32-jhk-36-5] Sainburg RL (2002). Evidence for a dynamic-dominance hypothesis of handedness. Exp Brain Res.

[b33-jhk-36-5] Savescu AV, Latash ML, Zatsiorsky VM (2008). A technique to determine friction at the fingertips. J Appl Biomech.

[b34-jhk-36-5] Seo NJ, Armstrong TJ, Chaffin DB, Ashton-Miller JA (2008). Inward torque and high-friction handles can reduce required muscle efforts for torque generation. Hum Factors.

[b35-jhk-36-5] Serrien DJ, Wiesendanger M (2001). A higher-order mechanism overrules the automatic grip-load force constraint during bimanual asymmetrical movements. Beha Brain Res.

[b36-jhk-36-5] Sternad D, Dean WJ, Schaal S (2000). Interaction of rhythmic and discrete pattern generators in single-joint movements. Hum Mov Sci.

[b37-jhk-36-5] Uno Y, Kawato M, Suzuki R (1989). Formation and Control of Optimal Trajectory in Human Multijoint Arm Movement - Minimum Torque-Change Model. Biol Cybern.

[b38-jhk-36-5] Uygur M, de Freitas PB, Jaric S (2010a). Effects of varying the load force range and frequency on force coordination in static manipulation. Neurosci Lett.

[b39-jhk-36-5] Uygur M, de Freitas PB, Jaric S (2010b). Frictional properties of different hand skin areas and grasping techniques. Ergonomics.

[b40-jhk-36-5] Uygur M, Jin X, Knezevic O, Jaric S (2012). Two-dimensional static manipulation tasks: does force coordination depend on change of the tangential force direction?. Exp Brain Res.

[b41-jhk-36-5] Westling G, Johansson RS (1984). Factors influencing the force control during precision grip. Exp Brain Res.

[b42-jhk-36-5] White O, McIntyre J, Augurelle AS, Thonnard JL (2005). Do novel gravitational environments alter the grip-force/load-force coupling at the fingertips?. Exp Brain Res.

